# Toripalimab Plus Paclitaxel and Carboplatin as Neoadjuvant Therapy in Locally Advanced Resectable Esophageal Squamous Cell Carcinoma

**DOI:** 10.1093/oncolo/oyab011

**Published:** 2022-01-28

**Authors:** Wenwu He, Xuefeng Leng, Tianqin Mao, Xi Luo, Lingxiao Zhou, Jiaxin Yan, Lin Peng, Qiang Fang, Guangyuan Liu, Xing Wei, Kangning Wang, Chenghao Wang, Sha Zhang, Xudong Zhang, Xudong Shen, Depei Huang, Huan Yi, Ting Bei, Xueke She, Wenguang Xiao, Yongtao Han

**Affiliations:** 1 Department of Thoracic Surgery, Sichuan Cancer Hospital and Research Institute, School of Medicine, University of Electronic Science and Technology of China (UESTC), Chengdu, People’s Republic of China; 2 School of Medicine, University of Electronic Science and Technology of China (UESTC), Chengdu, People’s Republic of China; 3 Department of Endoscopy Center, Sichuan Cancer Hospital and Research Institute, School of Medicine, University of Electronic Science and Technology of China (UESTC), Chengdu, People’s Republic of China; 4 Department of Pathology, Sichuan Cancer Hospital and Research Institute, School of Medicine, University of Electronic Science and Technology of China (UESTC), Chengdu, People’s Republic of China; 5 Department of Drug Clinical Trial, Sichuan Cancer Hospital and Research Institute, School of Medicine, University of Electronic Science and Technology of China (UESTC), Chengdu, People’s Republic of China; 6 The Medical Department, 3D Medicines Inc., Shanghai, People’s Republic of China; 7 Shanghai Junshi Biosciences Co., Ltd, Shanghai, People’s Republic of China

**Keywords:** esophageal squamous cell carcinoma, neoadjuvant therapy, toripalimab, chemotherapy

## Abstract

**Introduction:**

Immune checkpoint inhibitors (ICIs) are effective in the treatment of advanced esophageal squamous cell carcinoma (ESCC); however, their efficacy in locally advanced resectable ESCC and the potential predictive biomarkers have limited data.

**Methods:**

In this study, locally advanced resectable ESCC patients were enrolled and received neoadjuvant toripalimab (240 mg, day 1) plus paclitaxel (135 mg/m^2^, day 1) and carboplatin (area under the curve 5 mg/mL per min, day 1) in each 3-week cycle for 2 cycles, followed by esophagectomy planned 4-6 weeks after preoperative therapy. The primary endpoints were safety, feasibility, and the major pathological response (MPR) rate; the secondary endpoints were the pathological complete response (pCR) rate, disease-free survival (DFS), and overall survival (OS). Association between molecular signatures/tumor immune microenvironment and treatment response was also explored.

**Results:**

Twenty resectable ESCC patients were enrolled. Treatment-related adverse events (AEs) occurred in all patients (100%), and 4 patients (22.2%) experienced grade 3 or higher treatment-related AEs. Sixteen patients underwent surgery without treatment-related surgical delay, and the R0 resection rate was 87.5% (14/16). Among the 16 patients, the MPR rate was 43.8% (7/16) and the pCR rate was 18.8% (3/16). The abundance of CD8^+^ T cells in surgical specimens increased (*P* = .0093), accompanied by a decreased proportion of M2-type tumor-associated macrophages (*P* = .036) in responders upon neoadjuvant therapy. Responders were associated with higher baseline gene expression levels of CXCL5 (*P* = .03) and lower baseline levels of CCL19 (*P* = .017) and UMODL1 (*P* = .03).

**Conclusions:**

The combination of toripalimab plus paclitaxel and carboplatin is safe, feasible, and effective in locally advanced resectable ESCC, indicating its potential as a neoadjuvant treatment for ESCC.

**Clinical Trial registration:**

NCT04177797

## Teaser

This article reports on the safety, feasibility, and efficacy of toripalimab (a PD-1 antibody) combined with paclitaxel and carboplatin for locally advanced resectable esophageal squamous cell carcinoma in the neoadjuvant setting.

**Implications for Practice**

The safety, feasibility, and efficacy of neoadjuvant immunotherapy in locally advanced esophageal squamous cell carcinoma (ESCC) have limited data. Our prospective study showed that toripalimab plus paclitaxel and carboplatin is safe and feasible in locally advanced ESCC (*N* = 20), and is effective with a major pathological response of 43.8% and a complete pathological response of 18.8%. Moreover, the abundance of CD8+ T cells in the tumor immune microenvironment increased (*P* = .0093), accompanied by the reduced proportion of M2-type tumor-associated macrophages (*P* = .036) in responders upon neoadjuvant therapy. Responders were associated with higher baseline gene expression levels of CXCL5 (*P* = .03) and lower baseline levels of CCL19 (*P* = .017) and UMODL1 (*P* = .03). This study will provide useful information for neoadjuvant treatment of ESCC.

## Introduction

Esophageal squamous cell carcinoma (ESCC) is a common aggressive tumor that ranks as the 6th leading cause of cancer-related death worldwide.^1^ Most patients are already at a locally advanced stage when first diagnosed. In China, ESCC constitutes the predominant histology of esophageal cancer. Although recent studies reported that neoadjuvant chemoradiotherapy before surgery could significantly prolong overall survival (OS) and improve prognosis,^2,3^ a high risk of recurrence or metastasis still remains,^4,5^ and the 5-year OS rate is approximately 47%.^4,6^ Therefore, it is essential to find novel and effective treatment regimens for locally advanced resectable ESCC to further improve survival benefit.

Immune checkpoint inhibitors (ICIs), especially directed against programmed death-1 (PD-1) proteins, have indicated their safety and activity in various solid tumors.^7^ PD-1 pathway blockade provided insights into utilizing human autoimmunity against tumor cells and increased the antitumor immune response by reducing tumor clonal heterogeneity.^8^ The overexpression of PD-L1 was found in 48% of ESCC in tumor cells.^9^ Besides, based on whole-exome sequencing (WES) of tumor/blood samples, which revealed esophageal cancer cases exhibited high tumor mutation burden (TMB) values.^10^ The combining results indicated ESCC patients may potentially benefit from ICIs therapy. The randomized phase III KEYNOTE-181 study revealed that pembrolizumab (checkpoint inhibitor targeting PD-1) prolonged OS versus chemotherapy for advanced esophageal cancer in patients with PD-L1 combined positive score (CPS) > 10 in the second-line setting, with 18% of patients in the pembrolizumab group and 40.9% of patients in the chemotherapy group showed Grade 3 or higher treatment-related adverse events (AEs).^11^ Furthermore, compared with chemotherapy in previously treated patients with advanced ESCC, nivolumab (immune checkpoint PD-1 inhibitor) was associated with a significant improvement of OS in ATTRACTION-3 trial.^12^ In the KEYNOTE-590 trial where 73% of advanced esophageal cancer patients were squamous cell subtype, pembrolizumab combined with cisplatin-fluoropyrimidine chemotherapy could significantly improve the OS and progression-free survival (PFS) in biomarker selected subgroup of PD-L1 CPS ≥ 10 patients with ESCC, whereas this benefit did not appear in ESCC patients with PD-L1 CPS < 10 and adenocarcinoma patients (only PFS benefit).^13,14^ In the phase II RATIONALE 205 trial, which assessed the safety and efficacy of tislelizumab plus cisplatin and 5-Fu in unresectable ESCC patients, 46.7% of patients achieved an objective response.^15^ Taken together, the above results revealed ICIs have provided durable responses with acceptable safety in esophageal cancer patients.

In recent years, ICIs as neoadjuvant regimens have shown impressive and effective pathological responses for early-stage patients with non-small-cell lung cancer, melanoma, bladder cancer, and colon cancer^16-19^ with manageable treatment-related adverse effects. Currently, neoadjuvant immunotherapy expected to improve OS has been explored in esophageal cancer patients, and initial results are available. While preoperative PD-1 blockade combined with chemoradiotherapy induced a good pathological complete response (pCR) ratio for ESCC in recent phase II studies.^20,21^ Thus, it is worth exploring more options for immunotherapy combination regimens for ESCC in neoadjuvant therapy.

In this study, we investigated the safety, feasibility, and efficacy of toripalimab (a PD-1 antibody) combined with paclitaxel and carboplatin for locally advanced resectable ESCC in the neoadjuvant setting (NCT04177797). The features of pathological response, as well as the associations of response with absolute immune cell counts in peripheral blood, tumor genomic signatures, and the tumor immune microenvironment (TIME) were also investigated.

## Methods

### Patients

Eligible patients were 18-75 years of age and had histologically confirmed, potentially resectable ESCC with clinical stage III-IVa (T3-4aN1-3M0, AJCC 8th TNM classification); an Eastern Cooperative Oncology Group performance status score of 0 or 1; and normal organ function. Patients with a history of autoimmune disease; received systemic steroid therapy or any other immunosuppressive therapy; with active infection or virus infection; with a history of other immunotherapy or chemoradiotherapy; and unable to sign informed consent for any reason were excluded. This study was approved by the Ethical Committee of the Sichuan Cancer Hospital, China. All included patients signed an informed consent form (Ethics: KY-2019-041-01).

### Study Design

This study was a single-arm, open-label, phase II trial of neoadjuvant toripalimab combined with paclitaxel and carboplatin in locally advanced resectable ESCC. All included patients received toripalimab (240 mg, IV, D1), paclitaxel (135 mg/m2, IV, D1), and carboplatin (area under the curve [AUC] 5 mg/mL per min, IV, D1) in each 3-week cycle for 2 cycles before surgery. Minimally invasive McKeown esophagectomy was performed approximately 4-6 weeks after the second dose of preoperative treatment. The primary endpoints were safety, feasibility, and the major pathological response (MPR) rate, and the secondary endpoints were the pCR rate, disease-free survival (DFS), and OS. The correlations between molecular biomarkers including absolute immune cell counts in peripheral blood, genomic signatures and TIME, and the efficacy of neoadjuvant immunotherapy for ESCC were also explored.

During the neoadjuvant treatment period, all patients were monitored for AEs according to The Common Terminology Criteria for Adverse Events Version 4.03.^22^ All patients underwent baseline tumor staging, including neck, thorax, abdomen plain, and contrast-enhanced computed tomography (CT); esophagogastroduodenoscopy; ultrasound endoscopy (EUS); and cervical ultrasonography. If indicated, positron emission tomography-computed tomography (PET-CT) was performed to exclude distant metastatic disease. And then, the clinical staging of T/N in ESCC patient was evaluated according to AJCC 8th.^23^ Chest CT was repeated within a week before surgery, and the primary lesion was evaluated according to Response Evaluation Criteria in Solid Tumors (RECIST), version 1.1.^24^

### Pathological Assessment

All patients who underwent surgery had a final available pathological stage (ypTNM) after both primary tumors and lymph nodes were reviewed by experienced pathologists. The criterion of pathological assessment was according to the 8th edition of the TNM staging system.^25^ In addition, to objectively analyze the pathological features of the response to neoadjuvant treatment, resected specimens were independently re-evaluated by 2 experienced pathologists according to the criterion of immune-related pathological response.^26,27^ This criterion defined the tertiary lymphoid structure (TLS) as an ectopic, organized lymphoid node-like structure that includes T cells, B cells, and other immune cells and stroma.^26^ MPR was defined as having no more than 10% of residual viable tumor cells, and specimens that showed no evidence of vital residual tumor cells were defined as a pCR.

### WES and TIME by Multiplex Immunofluorescence (mIF)

To explore predictive biomarkers, gastroscopic biopsy tissues, including tumors and their adjacent normal tissues, and 15 mL of peripheral blood from each enrolled patient were collected before the first dose. All tissues were immediately processed as formalin-fixed, paraffin-embedded (FFPE) samples for testing. The pretreatment tumor FFPE samples were subjected to WES with an average sequencing depth of 345× (range 262-445×). Multiplex immunofluorescence staining was conducted using the PANO 7-plex IHC kit (Panovue). Primary antibodies targeting CD8 (clone C8/144B), CD56 (clone 123C3), HLA-DR (clone EPR3692), CD68 (clone BP6036), and PanCK (cocktail) were sequentially applied to FFPE tissue slides. FFPE tissue sections were subjected to assess PD-L1 expression by using the PD-L1 IHC 22C3 pharmDx assay (Agilent Technologies). PD-L1 expression was determined using the tumor proportion score (TPS), which is the proportion of viable tumor cells showing partial or complete membrane PD-L1 staining at any intensity. PD-L1 expression was also defined using the CPS by dividing the number of PD-L1-stained cells (tumor cells, lymphocytes, and macrophages) by the total number of viable tumor cells and multiplying by 100.

### Statistical Analysis

For biomarker analysis, the significance of categorical variables (eg, PD-L1 status) was assessed by Fisher’s exact test. Continuous variables that conformed to a normal distribution were assessed by Student’s *t*-test. If the data failed to meet a normal distribution, nonparametric analyses, that is, the Mann–Whitney *U* test and Wilcoxon’s rank-sum test, were used. All of the above tests were 2-sided, and statistical significance was set to a *P* value of less than .05. The statistical analysis was conducted using R (version 4.0.2).

## Results

### Patient Characteristics

**Figure 1.**
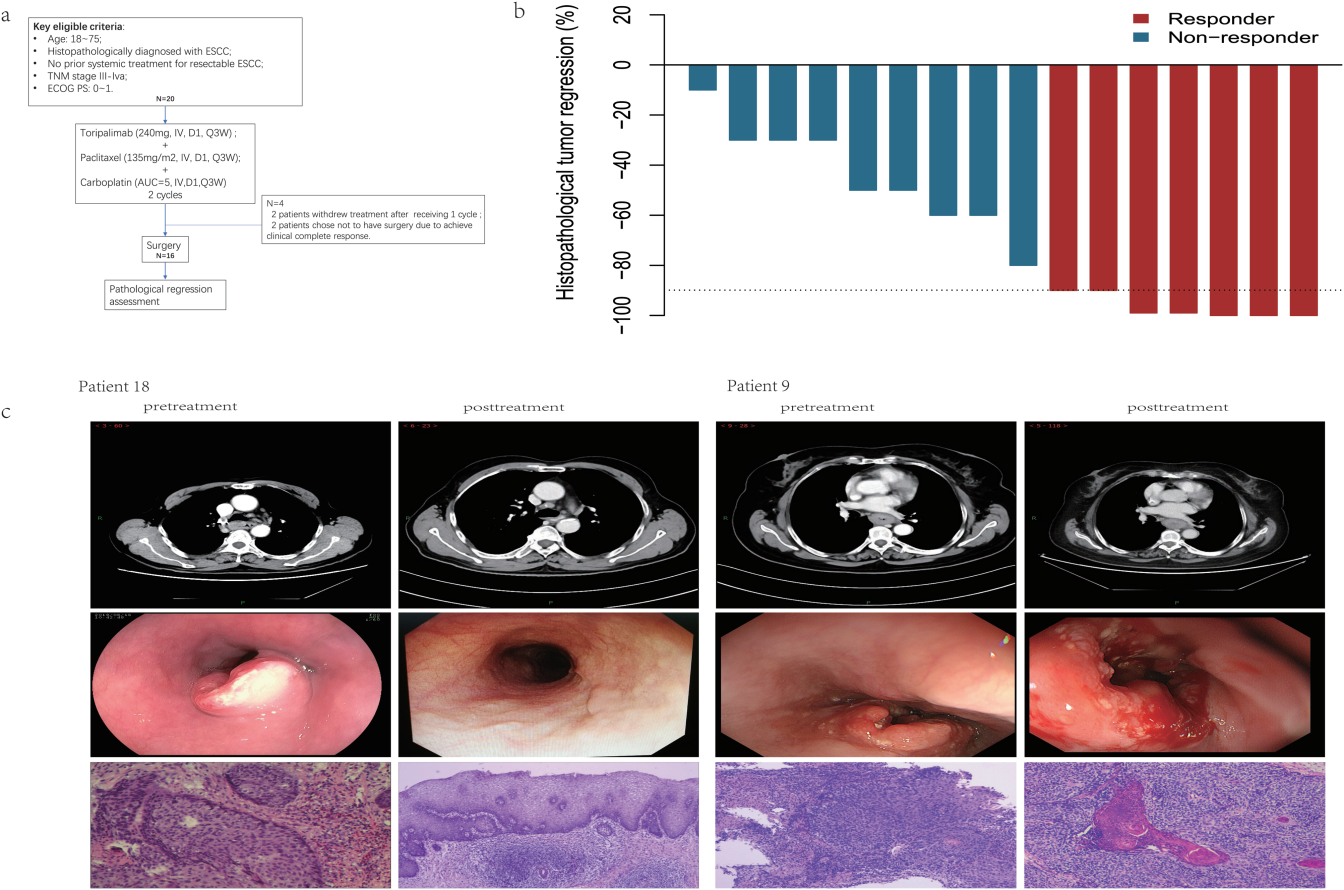
(**a**) Patient enrollment overview. ESCC, esophageal squamous cell carcinoma. (**b**) Percentage pathological regression after neoadjuvant immunotherapy and chemotherapy shown per tumor. The horizontal dotted line depicts the demarcation for major pathological responses with 90% tumor regression. (**c**) Cases of radiological and pathological response after neoadjuvant immunotherapy and chemotherapy. The vertical black line separates patients with good efficacy (left) and stable disease (right). (i) Top left: CT imaging of the chest of a 68-year-old man with stage III/T3N1M0, middle third before treatment. Top right: Posttreatment CT scan showed no notable disease. Middle row: pre- and posttreatment endoscopic pictures of the tumor. Bottom: pre- and posttreatment pathological assessment with microscopy by HE staining. (ii) Top left: CT imaging of the chest of a 62-year-old woman with stage III/T3N2M0, middle third before treatment. Top right: Posttreatment CT scan showing notable disease. Bottom: pre- and posttreatment pathological assessment with microscopy by HE staining.

A total of 20 patients were enrolled from January 2020 to June 2020. Two patients refused to subsequent treatment after receiving 1 cycle of dose, 2 patients refused to surgery for achieving complete clinical response after preoperative treatment, and 16 patients completed continuity treatment (Figure 1a). Among them, 80% (16/20) of patients had stage III disease, 70% (14/20) of patients were current or former smokers, and 65% (13/20) of patients had a drinking history (Table 1).

**Table 1.** Characteristics of patients at baseline based on pathological response.

Characteristics	All patients *N* = 20	Patients with major pathological response *N* = 7	Patients without major pathological response *N* = 9	*P*-value
Age				.134
Mean ± SD	61.4 ± 6.5	64.0 ± 7.4	58.8 ± 5.7	
Median (range)	62.1 (51.5-72.3)	66.6 (51.5-72)	58 (51.5-67.3)	
Sex				.308
Female	5 (25.0%)	1 (14.3%)	4 (44.4%)	
Male	15 (75.0%)	6 (85.7%)	5 (55.6%)	
Smoking	14(70.0%)	5 (71.4%)	5 (55.6%)	.633
Drinking	13 (65.0%)	4 (57.1%)	5 (55.6%)	1.000
Clinical stage				1.000
III	16 (80.0%)	6 (85.7%)	7 (77.8%)	
IVa	4 (20.0%)	1 (14.3%)	2 (22.2%)	
Tumor location				.550
Middle third	14 (70.0%)	5 (71.4%)	8 (88.9%)	
Lower third	6 (30.0%)	2 (28.6%)	1 (11.1%)	
Tumor differentiated^a^				.122
Well differentiated	1 (5.6%)	-	1 (11.1%)	
Moderate differentiated	5 (27.8%)	1 (14.3%)	4 (44.4%)	
Poor differentiated	6 (33.3%)	2 (28.6%)	4 (44.4%)	

There were 8 patients without pathological assessment of differentiated due to 4 patients refusing surgery, and 4 patients without pathological assessment due to their achieved pathological complete response.

### Safety and Feasibility

No unreported toxicity was observed during neoadjuvant treatment period. Treatment-related AEs occurred in all patients (20/20, 100%), the most frequent AEs were nausea (15/20, 75%), anemia (14/20, 70%), alopecia (9/20, 45%), leukopenia (8/20, 40%), and vomit (6/20, 30) (Table 2). Four patients (4/20, 20.0%) experienced grade 3 or higher treatment-related AEs, including neutropenia (2/20, 10.0%), leukopenia (1/20, 5.0%), and fatigue (1/20, 5.0%). No treatment-related deaths occurred (Table 2).

**Table 2.** Adverse events during immunotherapy combined chemotherapy.

Adverse events	Number of events (%)		
	Grade 1/2	Grade 3	Grade 4
Any adverse events	16 (80.0%)	1 (5.0%)	3 (15.0%)
Blood			
Anemia	14 (70.0%)	-	-
Leukopenia	8 (40.0%)	1 (5.0%)	-
Neutropenia	3 (15.0%)	-	2 (10.0%)
Thrombocytopenia	4 (20.0%)	-	-
Gastrointestinal			
Nausea	15 (75.0%)	-	-
Vomit	6 (30.0%)	-	-
Diarrhea	5 (25.0%)	-	1 (5.0%)
Constipation	3 (15.0%)	-	-
Colitis	2 (10.0%)	-	-
Dermatitis	2 (10.0%)	-	-
Fatigue	5 (25.0%)	1 (5.0%)	-
Alopecia	9 (45.0%)	-	-

Two patients (2/20) who refused to subsequent treatment after receiving 1 cycle of dose were excluded from the pathological evaluation and effectiveness analysis. In total, 18 patients (18/20) completed 2 cycles of neoadjuvant therapy. Two patients (2/20) chose not to undergo surgery for achieving clinical complete response (cCR) after preoperative treatment. It is worth noticing that these 2 patients’ tissues were obtained after neoadjuvant treatment through gastroscopic biopsy at the location of the primary tumor, and no residual viable tumor cells were observed. Thus, these 2 patients (2/20) were included for the evaluation of effectiveness but not for the evaluation of tumor regression. The remaining 16 patients (16/20) who underwent surgery were without treatment-related surgical delay and evaluated for tumor regression evaluation and effectiveness. The median duration between the administration of the first dose of drugs and surgery was 61.5 (range 55-71) days. In this surveillance interval, no anastomotic leakage or other surgery-related AE was observed.

### Imaging Assessment

The median duration of imaging assessment for disease by CT scan was 56.5 days (50-66 days) after receiving the first doses. All patients underwent radiologic response evaluation, and representative images are shown in Figure 1c. Among the 18 patients, 6 patients (6/18, 33.3%) had a complete response, 5 patients (5/18, 27.8%) had a partial response, 7 patients (7/18, 38.9%) had stable disease (SD), and none of the patients had progressive disease (PD). Pathological downstaging from the pretreatment clinical stage occurred in 13 patients (13/18, 72.2%) (Table 3).

**Table 3:** Pretreatment clinical stage and posttreatment pathological stage.

Patients’ ID	cTNM	Response	ypTNM	Pathological response	R0 resection	Downstaging
SC01-001	T3N1 (III)	PR	T1N0 (I)	Major response	Yes	Yes
SC01-002	T3N2 (III)	CR	-	NA	NA	No
SC01-003	T3N1 (III)	CR	T0N0 (I)	Major response	Yes	Yes
SC01-004	T3N2 (III)	SD	T3N1 (IIIB)	<90%	Yes	Yes
SC01-005	T3N1 (III)	PR	T3N0 (II)	<90%	Yes	Yes
SC01-006	T4aN3 (IVA)	SD	T3N2 (IIIB)	<90%	No	Yes
SC01-007	T3N2 (III)	PR	T2N0 (I)	Major response	Yes	Yes
SC01-008	T4aN1 (IVA)	SD	T2N0 (I)	<90%	No	Yes
SC01-009	T3N2 (III)	SD	T3N0 (II)	<90%	Yes	Yes
SC01-010	T3N2 (III)	CR	-	NA	NA	No
SC01-011	T3N1 (III)	SD	T3N1 (IIIB)	<90%	Yes	No
SC01-012	T3N3 (IVA)	PR	T1N0 (I)	Major response	Yes	Yes
SC01-013	T3N1 (III)	CR	TisN0 (I)	Complete response	Yes	Yes
SC01-014	T3N1 (III)	CR	T0N0 (I)	Complete response	Yes	Yes
SC01-015	T3N2 (III)	PR	T3N1 (IIIB)	<90%	Yes	Yes
SC01-016	T3N1 (III)	SD	T3N2 (IIIB)	<90%	Yes	No
SC01-017	T3N1 (III)	SD	T3N1 (IIIB)	<90%	Yes	No
SC01-018	T3N1 (III)	CR	T0N0 (I)	Complete response	Yes	Yes

Abbreviations: CR, complete response; PR, partial response; SD, stable disease.

### Pathological Features

R0 resection rate was achieved for 87.5% (14/16) who received surgery. Two patients (2/16) who diagnosed with clinical T4a stage before treatment were assessed as having SD by imaging after treatment and underwent R1 resection. Among the 16 patients with evaluable specimens, 7 (7/16, 43.8%) patients achieved MPR, and 3 (3/16, 18.8%) patients achieved pCR (Figure 1b). The lymph nodes of all MPR patients were negative by pathological evaluation.

In addition, the TLSs in all resection samples/gastroscopic biopsy after neoadjuvant therapy were investigated. We found that 11 patients (11/18, 61.1%) had TLSs, and most of them (6/11, 54.5%) achieved MPR. In contrast, among the 5 patients’ samples without TLSs, 4 patients (4/5, 80%) had numerous residual tumor cells. However, this difference between the 2 groups was not statistically significant (*P* = .308).

### Genomic Analysis

**Figure 2.**
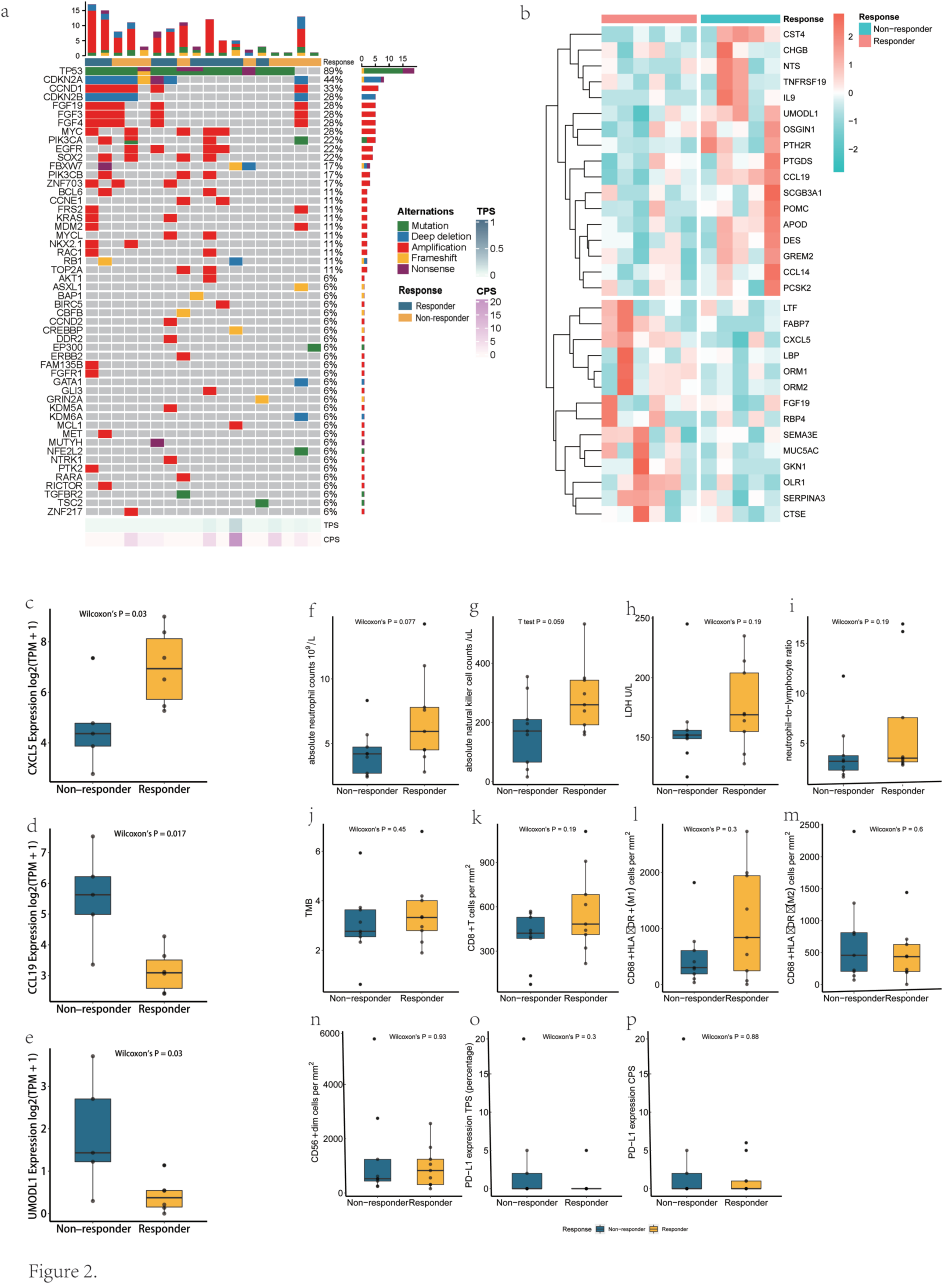
(**a**) The landscape of genomic alterations in baseline tissue samples of 18 patients with ESCC undergoing treatment. (**b-e**) Immune gene signatures and differences between the responders and nonresponders in 31 immune-related genes expression. (b) Immune gene signatures according to response. (c) Differences between the responders and nonresponders in CXCL5 expression. (d) Differences between the responders and nonresponders in CCL19 expression. (e) Differences between the responders and nonresponders in UMODL1 expression. (**f-p**) Comparisons between responders and nonresponders before treatment for subsets of biomarkers. (f) Absolute neutrophil counts. (g) Absolute natural killer cell counts. (h) Lactate dehydrogenase. (i) Neutrophil-to-lymphocyte ratio. (j) TMB. (k) CD8+ T cells density. (l) CD68+HLA-DR+ density. (m) CD68+HLA-DR- density. (n) CD56+dim density. (o) PD-L1 expression TPS. (p) PD-L1 expression CPS. ESCC, esophageal squamous cell carcinoma; TMB, tumor mutation burden; TPS, tumor proportion score.

WES from pretreatment samples showed that all 18 patients who completed 2 cycles of neoadjuvant therapy carried deleterious somatic variations, most of which were single-nucleotide variants (SNVs, 16/18, 89%) and copy number variations (CNVs, 12/18, 67%) (Figure 2a). TP53 was the most commonly mutated gene (16/18, 89%), and other frequently mutated genes in more than 4 patients were CDKN2A (8/18, 44%), CCDN1 (6/18, 33%), MYC (5/18, 28%), FGF3 (5/18, 28%), FGF4 (5/18, 28%), FGF19 (5/18, 28%), and CDKN2B (5/18, 28%). All pretreatment samples were microsatellite stable (MSS) tumors, with a mean TMB value of 3.28 ± 1.40 muts/Mb. Among them, there were 4 patients (22%) with a TPS of PD-L1 expression greater than 1% and 7 (39%) patients with a CPS of PD-L1 expression greater than 1.

### Definition of Responders and Nonresponders After Neoadjuvant Immunotherapy

To further investigate potential biomarkers that can predict the tumor response, we divided the patients who completed the preoperative therapy into responders and nonresponders. First, we classified the responders and nonresponders according to pathological response rate. Among the 16 patients who underwent surgery, 7 achieved MPR and 9 had non-MPR with more than 10% residual viable tumor. Second, 2 patients who did not undergo surgery had cCR confirmed by CT and no residual viable tumor in their gastroscopic biopsy tissues was observed after neoadjuvant therapy. We regarded these 2 patients as having a good response. Thus, we defined the responders as either patients who achieved MPR after surgery or patients who had cCR without surgery (total *N* = 9), and the rest of the patients were defined as nonresponders (*N* = 9).

### Biomarker Analysis Between Responders and Nonresponders in ESCC Patients Received Neoadjuvant Immunotherapy

To evaluate the relationship between systemic inflammation and the efficiency of immunotherapy, pretreatment peripheral blood data (including absolute natural killer cell counts, absolute neutrophil counts [ANC], neutrophil-to-lymphocyte ratio [NLR] and lactate dehydrogenase [LDH]) were collected from 18 patients. The analysis results revealed that ANC (*P* = 0.077) and absolute natural killer cell counts (*P* = 0.059) tended to be higher in responders than in nonresponders (Figure 2f-g). Certain blood markers, such as LDH and NLR, successfully predicted the outcome of immunotherapy in some tumors in previous literature;^28^ however, they were not related to tumor response in this study (*P* = .19 and .19, respectively, Figure 2h and i).

Since TMB and tumor-infiltrating lymphocytes (TILs) are also regarded as potentially predictive markers in immunotherapy, we obtained the TMB values of 18 patients from each WES result and TIL densities from mIF (Figure 2j). However, TMB failed to divide the 2 groups significantly (*P* = .45). Additionally, there were no significant differences of CD8+ (*P* = .19), CD68+HLA-DR+ (M1-TAM, *P* = .30), CD68+HLA-DR- (M2-TAM, *P* = .60), and CD56+dim (*P* = .93) TIL densities between responders and nonresponders (Figure 2k-n). Furthermore, neither the TPS nor CPS of PD-L1 expression could predict the tumor response (*P* = .3 and .88, respectively, Figure 2o and p).

To further investigate possible biomarkers, we collected gene expression data of tumor tissues from 11 patients before neoadjuvant treatment and explored the genomic difference between responders and nonresponders. The analysis showed that there was a total of 31 immune-related genes that had significant differences in gene expression (Figure 2b). In particular, responders had higher chemokine CXCL5 (*P* = .03) expression and lower chemokine CCL19 (*P* = .017) and UMODL1 (*P* = .03) expression compared with nonresponders (Figure 2c-e).

### Dynamic Changes in the TIME Before and After Neoadjuvant Immunotherapy

**Figure 3.**
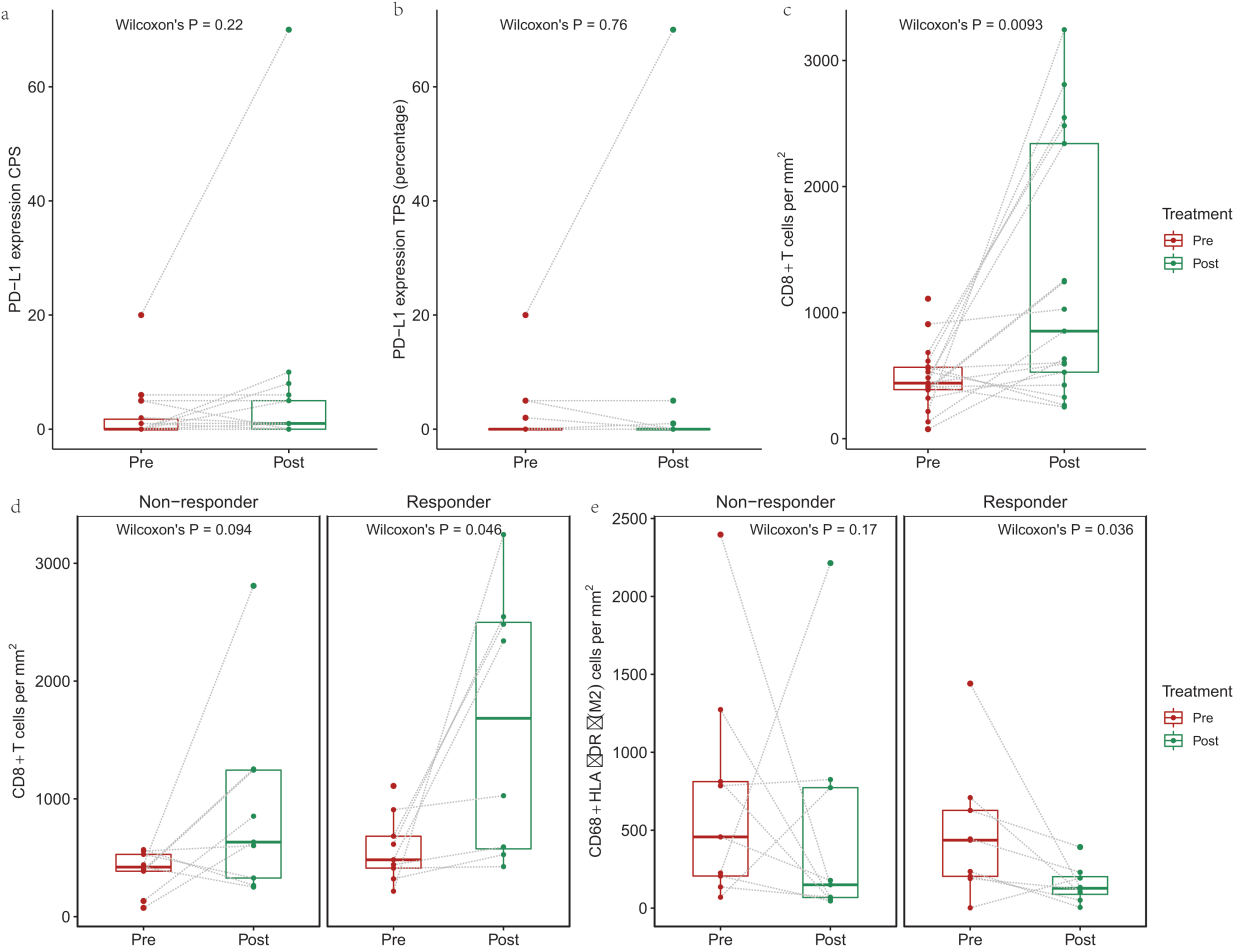
Differences in TIME between pre- and posttreatment, and pre- to posttreatment changes in responders and nonresponders. (**a**) Differences between pre- and posttreatment in PD-L1 expression TPS. (**b**) Differences between pre- and posttreatment in PD-L1 expression CPS. (**c**) Differences between pre- and posttreatment in CD8+ T cells density in 17 pared samples. (**d**) Differences between pre- and posttreatment in CD8+ T cells density in nonresponders (left) and in responders (right). (**e**) Differences between pre- and posttreatment in CD68+HLA-DR- density in nonresponders (left) and in responders (right). CPS, combined positive score; TIME, tumor immune microenvironment; TPS, tumor proportion score.

To explore the changes in the TIME after neoadjuvant immunotherapy, the mIF data of paired tissue samples before and after neoadjuvant immunotherapy from 17 patients were collected (including responders, *N* = 8; nonresponders, *N* = 9). PD-L1 expression, CD8+, M1-TAM, M2-TAM, and CD56+dim TIL densities in 17 paired tissue samples were investigated (Figure 3). The CPS of PD-L1 expression was decreased in 3 patients (3/17, 18%) but increased in 6 patients (6/17, 35%), including 5 patients whose CPS changed from 0 to ≥ 1 (5/6, 83%). TPS did not significantly change, with 2 patients each showing increased and decreased values (12%). Both of 2 PD-L1 expression values (TPS and CPS) showed no statistical difference before and after treatment (Figure 3a and b). CD8+ TIL density significantly increased (*P* = .0093) after neoadjuvant combined immunotherapy (Figure 3c). This significant increase in CD8+ TIL density appeared in the responders (*P* = .046; Figure 3d, right) but not in the nonresponders (*P* = .094; Figure 3d, left). Moreover, density of M2-TAM TIL significantly decreased in responders (*P* = .036; Figure 3e, right) after neoadjuvant treatment, whereas no significant difference was observed in nonresponders (*P* = .17; Figure 3e, left).

## Discussion

In this study, we presented that preoperative administration of 2 cycles of toripalimab combined with paclitaxel and carboplatin led to 43.8% MPR and 18.8% pCR for locally advanced resectable ESCC. The toripalimab-related AEs were consistent with those reported in previous studies; in this study, the dominant immune-related AE was dermatitis and colitis.^29,30^ Any other grade AEs were in line with chemotherapy-related AEs, including common hematologic toxicities and gastrointestinal discomfort.^3^ Toxicity compared with triplet treatment in CROSS trial and NEOCRTEC5010 trial, no new chemotherapy-related AEs were observed in this study. The baseline characteristics of patients between our study and CROSS, NEOCRTEC5010 were compared, we found the age of patients in our study was similar in CROSS, both of them were older than patients in NEOCRTEC5010. In total, the rate of AEs was higher in our study than CROSS trial, lower than NEOCRTEC5010 trial. In the case of no comorbidity in patients were reported in these 3 trials, larger sample size and randomize control needed to conduct to investigate the change of AEs after immunotherapy treatment combined with chemotherapy. In this study, the results showed that the combination of toripalimab plus paclitaxel and carboplatin is safe and feasible with manageable treatment-related AEs and without surgery delay in locally advanced ESCC.

Currently, standard treatments for resectable locally advanced esophageal cancer mainly include neoadjuvant chemoradiotherapy (NCRT) and neoadjuvant chemotherapy (NCT) before surgery. The results of CROSS^31^ and NEOCRTEC5010^32^ trials confirmed that neoadjuvant chemoradiotherapy (NCRT) plus surgery could improve survival over surgery alone for locally advanced esophageal or esophagogastric junctional cancer, with acceptable and controllable safety. Thus, the combination of NCRT with surgery resection is the recommended treatment approach by guidelines and clinical practice for locally advanced EC patients in most Western and Asian (China and South Korea) countries.^33^ Several clinical trials including the MAGIC study, have also shown that NCT plus surgery offered a superior survival benefit compared with surgery alone for resectable thoracic esophageal cancer patients.^34,35^ In the past decades, NCT has constituted the mainstay of treatment for locally advanced stages esophageal cancer in Asia, especially in Japan.^36^ Furthermore, the results from several randomized clinical trials and retrospective studies in neoadjuvant therapy of advanced esophageal cancer revealed that compared with NCT, NCRT resulted in better histopathologic outcome, higher R0 resection rate, more successful in downstaging and a higher frequency of negative lymph nodes, however, no significantly improved long-term survival was observed.^37-39^ In general, a combination of NCRT or NCT with surgery resection has improved survival for resectable locally advanced esophageal cancer patients, but the prognosis of these set of patients is still unsatisfactory.

Recently, immunotherapy with ICIs targeting the PD-1/ PD-L1 pathway has shown positive results for advanced EC patients in the studies (KEYNOTE-181, ATTRACTION-3, and KEYNOTE-590, etc.). Several clinical trials have started to attempt the immunotherapy combined with chemoradiotherapy/chemotherapy in the neoadjuvant setting for locally advanced esophageal cancer (PALACE-1 trial, PERFECT trial, NCT04177875, NCT03946969, NCT03200691, et al) to explore whether the addition of ICIs can improve OS benefit. A 55.6% pCR rate was achieved with the combination of pembrolizumab and chemoradiotherapy in the PALACE-1 study, which was slightly higher than the 49% pCR rate in the ESCC subgroup of CROSS trial^40^ and 43.2% pCR rate in the NEOCRTEC5010 trial,^32^ however, the long-term survival needed further evaluated. In the PERFECT trial, the feasibility and efficacy of nCRT combined with atezolizumab (a PD-L1 inhibitor) for resectable esophageal adenocarcinoma was investigated, with a pCR rate was 25%. In addition, a statistically significant difference in survival between the PERFECT and the NCRT cohort was not observed.^41^ In the KEYNOTE-590 trial, pembrolizumab plus chemotherapy as first-line therapy obtained promising results in advanced esophageal cancer, more novel treatment options are needed to evaluate the efficiency and the survival outcomes of locally advanced esophageal cancer. In this study, we considered paclitaxel and carboplatin as the combined regimen according to the CROSS clinical trial. Based on the NEOCRTEC5010, KEYNOTE 180,^42^ and KEYNOTE 181^11^ studies, the administration of the combined regimen was 2 cycles every 3 weeks, and the duration between preoperative treatment and surgery was arranged in 4-6 weeks. In our study, although the pCR rate was 18.8%, which was lower than the reported pCR rate in CROSS and NEOCRTEC5010 trials, the long-term clinical benefit was unknown, and DFS and OS data were needed to further investigate. Increasing the number of treatment cycles might improve the pCR or MPR rates, which is worthy of further exploration.

In this study, the results showed that the response status was different between imaging assessment and pathological assessment. This may be evidence of fibroplasia or inflammatory reactions instead of the tumor.^19^ We also found that MPR patients had a complete response in lymph nodes. We consider that the mechanism of immunotherapy is through activating the immune reaction, and the critical place is the lymph node in the body. Therefore, we hypothesized that lymph nodes may be a predictor of response to neoadjuvant immunotherapy.

In our study, we also found that the existence of TLSs was associated with notable tumor responses. TLSs could improve antigen presentation and increase cytokine-mediated signaling, which leads to improved prognosis after immunotherapy.^43^ A recent study reported that a dysfunctional T-cell microenvironment exists without TLSs.^44^ The change in the microenvironment after immunotherapy is complex and crucial. Although our results showed that TLSs were related to the tumor response, the specific mechanism, and its function in the microenvironment needs to be further analyzed.

For the combined regimen of PD-1 blockade and chemotherapy drugs, biomarkers’ predictive capacity might be weakened. In this study, we attempted to identify “well reported” immune-related biomarkers, such as PD-L1 expression, TMB, and TILs. However, most of the markers did not predict the response to immunotherapy between responders and nonresponders, and we observed that the absolute counts of neutrophils and natural killer cells in peripheral blood tended to be higher in responders. One possible explanation for this result is that the combination therapy reduces the predictive power of the markers. Previous research results have shown that chemotherapy drugs can increase CD8+ TIL density,^45^ regulate the immune microenvironment,^46^ and upregulate the expression of PD-L1 in ESCC tumors,^47,48^ that may promote cancer cell susceptibility to immune therapy. Although CPS in the KEYNOTE-181 trial,^11^ Tim 3+ T cells,^49^ or LDH levels in peripheral blood in small sample studies^50^ have been proven to predict the efficacy of immunotherapy in advanced ESCC, this trial was immune monotherapy instead of combination therapy. For most studies on combined regimens, such as PALACE-1^21^ and NICE^51^ trial, biomarkers could not predict the tumor response or survival, except for KEYNOTE 590 trial.

TIME plays an important role in tumor progression, tumor response to treatment, and drug resistance. Dynamic changes of infiltrating immune cells after neoadjuvant treatment are limited reported, especially for ESCC. In a previous study, platinum- and taxane-based neoadjuvant chemotherapy was associated with increased densities of CD3+, CD8+, CD8+ TIA-1+, PD-1+, and CD20+ TIL in ovarian cancer.^52^ In this study, we explored the dynamic changes of PD-L1 expression and TILs in tumors before and after neoadjuvant therapy. The CD8+ TIL density increased significantly after neoadjuvant immunotherapy for the responders; in contrast, no significant increased CD8+ TIL density was observed in nonresponders. In addition, we also found that M2-TAM TIL density decreased significantly in responding patients. It is worth noting that, CXCL5 and CCL19 gene expression, which were related to TAMs, showed a significant difference between the responders and the nonresponders. CXCL5, which releases from M2-TAMs, promotes an immunosuppressive tumor microenvironment^53^; CCL19 is a critical regulator in immune surveillance^54^ and proved to induce M1-TAMs chemotaxis but not M2-TAMs.^55^ Meanwhile, TAMs express PD-1 and PD-1/L1 blockade could effect on them to reduce tumor growth.^56^ Therefore, the effective immune response in this study may be related to the release of M2-TAMs immunosuppression after PD-1 antibody therapy, which required further investigation. In addition, the number of patients with TPS ≥ 1% remained the same after combined therapy, which was inconsistent with a previous study showing that chemotherapy can upregulate TPS expression.^45^ Conversely, 35% of the post-treatment samples showed an increase in CPS. This phenomenon was also observed in a previous study, which indicated that neoadjuvant chemotherapy (5-fluorouracil and cisplatin) induced PD-L1 expression more significantly on immune cells than tumor cells in ESCC.^45^

The main limitation of our study is the limited sample size. Additionally, the follow-up duration after surgery was relatively short, whether the long-term clinical benefit is related to pathological response requires further DFS follow-up data to support.

In conclusion, the preoperative combination of toripalimab, paclitaxel, and carboplatin for patients with locally advanced resectable ESCC was well tolerated and showed antitumor activity, indicating this combination would be a potential neoadjuvant therapy regimen for locally advanced resectable ESCC. Furthermore, the immune-related genes of CCL19, CXCL5, and UMODL1 might be potential predictors for immunotherapy in the neoadjuvant setting for ESCC, but which still need to be further confirmed. Neither TMB nor PD-L1 expression in pretreatment tissue would be able to predict tumor response. Further studies with a larger sample size are needed to conduct.

## Data Availability

The data underlying this article will be shared on reasonable request to the corresponding author.
